# One-Pot Green Preparation of Fluorescent Cellulose Nanofibers

**DOI:** 10.3390/polym14071313

**Published:** 2022-03-24

**Authors:** Qilin Lu, Jiayin Wu, Hanchen Wang, Biao Huang

**Affiliations:** 1 Fujian Key Laboratory of Novel Functional Textile Fibers and Materials, Minjiang University, Fuzhou 350108, China; wjymdcg@163.com (J.W.); wanghc08@163.com (H.W.); 2 College of Material Engineering, Fujian Agriculture and Forestry University, Fuzhou 350002, China; bhuang@fafu.edu.cn

**Keywords:** fluorescent cellulose nanofibers, high yield, one-pot, green preparation, chloride ion detection

## Abstract

Fluorescent cellulose nanofibers (FCNFs), with a high yield, were prepared via one-pot hydrolysis and the grafting reaction of cellulose with thiazolipyridine carboxylic acid (TPCA). The hydrolysis and Fischer esterification of cellulose were conducted under microwave-hydrothermal conditions; meanwhile, TPCA formation was induced by the dehydration reaction between L-cysteine and citric acid. The effects of the reaction temperature and reaction time on the yield and performance of FCNF were investigated. The morphology and size, surface chemical property, crystal structure, thermostability, and fluorescent performance of FCNF were characterized. The results revealed that the yield of FCNF reached 73.2% under a microwave power of 500 W, reaction temperature of 110 °C, and reaction time of 5 h. The FCNF obtained presents a short rod-like morphology. The crystallinity of the FCNFs is 80%, and their thermal stability did not decline significantly. Additionally, the fluorescent performance of the FCNFs is excellent, which results in them having good sensitivity to chloride ions. The good fluorescent performance and significant responsiveness to chloride ions of FCNFs lead to them having broad prospects in bio-labeling, biosensing, information storage, chloride ion detection, among others.

## 1. Introduction

As one of the representative products of cellulose-based nanomaterials, cellulose nanofibers (CNFs) have gained wide attention from researchers, due to their unique properties. Compared with native cellulose or microcrystalline cellulose, CNF has been recognized to possess numerous merits, such as a high specific surface area, high crystallinity, high purity, high Young’s modulus, biodegradability, easy functional modification, and good biocompatibility, as well as being natural and renewable [1,2,3]. In addition, nanocellulose exhibits nano-particle specificity, due to its nano effect, so it has broader application prospects in the fields of optoelectronic materials, sensing devices, and smart materials [4,5,6,7,8,9,10,11,12]. By means of functional modification, more functional cellulose-based nanomaterials can be designed, that is, nanocellulose can be used as a raw material to prepare fine chemicals and composite materials with special functions [13,14,15,16]. Therefore, how to prepare nanocellulose-based functional materials by a green and efficient method is a critical issue in the application field of nanocellulose, which is of great research significance to realize the high-value utilization of cellulose-based materials.

There are abundant hydroxyl groups in cellulose chains to provide many reactive sites for incorporating fluorophores into the cellulose backbone, resulting in diverse cellulose-based fluorescent nanomaterials [17,18]. The strategies for fabricating fluorescent nanocellulose mainly include chemical methods and physical adsorption. Compared with chemical modifications, physical adsorption has the advantages of simplicity, time saving, and maintaining the nanocellulose nature, due to the absence of solvent exchange. However, its application is limited by the properties of fluorophores and the low structural stability of the product. Accordingly, the drawbacks to chemical methods are the cumbersome processes associated with the separation of hydrolysis and modification, and environmentally unfriendly preparation, with the extensive use of organic solvents and large energy consumption. Chemical methods mainly involve carbodiimide coupling chemistry, Fischer–Speier esterification reactions, and stepwise activation functionalization [19,20,21,22]. Among these, the Fischer–Speier esterification reaction is regarded as the most facile method, with a high modification density, good dispersion stability, and retention of the cellulose physical structure, although the low yield of product is still a major shortcoming. Herein, the intervention of mechanochemistry aims to improve the yield of fluorescent nanocellulose, simplify the process, and reduce the energy consumption for fabrication.

It was found that the dehydration reaction of citric acid and L-cysteine can take place at high temperature to form the conjugated structure of thiazolipyridine carboxylic acid (TPCA), which has good fluorescence properties and biocompatibility. The main spectral transition of TPCA is derived from the conjugated 2-pyridone structure, resulting in the bright fluorescence of TPCA-modified fluorescent materials [23,24,25]. At a certain pH value, Cl^-^ has a dynamic quenching ability for TPCA, because the electronegative elements of N and -C=O in the TPCA structure are in an internal charge transfer state during excitation, which is conducive to the resonance of the enol. Furthermore, this excited state enhances the transition dipole moment of TPCA and the properties of sp^3^ on -C=O, increasing its absorption and emission intensity, resulting in the loss of planarity and rigidity of the conjugated 2-pyridone system, and causing out-of-plane vibrations to enter the non-radiative relaxation channel, which is the critical principle of the fluorescence quenching process of TPCA by chloride ions [26,27,28,29,30,31]. At present, TPCA-modified fluorescent materials have been widely used in biological imaging, sensors, drug delivery, fluorescence detection, and other fields [32,33,34,35,36].

In this study, the microwave-hydrothermal one-pot method was adopted to prepare FCNF. Hydrolysis and Fischer esterification of cellulose occur with citric acid to form cellulose nanofibers, which undergo a dehydration reaction with L-cysteine to form the TPCA structure in situ simultaneously, endowing FCNF with fluorescence properties. The whole preparation process is carried out in the aqueous phase and avoids the use of organic solvents, so as to be environmentally friendly. Moreover, the separation steps of the intermediate products and the generation of by-products are reduced by the one-pot method, which is conducive to improving the yield of the target product, and provides a new idea for the green and efficient preparation of fluorescent cellulose nanofibers. The fluorescent properties, biodegradability, and biocompatibility of FCNF have exhibited a broad range of application prospects in the fields of biomarkers, information storage, and sensing detection.

## 2. Experimental

### 2.1. Materials

The cellulose was obtained from bleached eucalyptus kraft pulps (BEKP) with a kappa number of 17 and an α-cellulose content greater than 94%, purchased from Fujian Nanping Paper Mill, Fujian, China. Citric acid and L-cysteine were supplied by Shanghai Aladdin Biochemical Technology Co., Ltd., Shanghai, China. All reagents were of analytical grade and were used without further purification.

### 2.2. Preparation of Fluorescent Cellulose Nanofibers

First, 3 g of BEKP, 15 g of citric acid and 30 mL of deionized water were added to a hydrothermal reactor, and the reaction was allowed to progress at 90–130 °C for 1 h with a microwave power of 500 W. Then, 0.8 mol/L L-cysteine was added to the reactor for 2–6 h under the same microwave-hydrothermal reaction conditions. After cooling to room temperature naturally, the product was centrifuged and washed with deionized water repeatedly until the supernatant had no fluorescence absorption under UV–visible light. The light yellow suspension on the upper layer was collected, namely, FCNF. The effects of temperature and reaction time on the yield and fluorescence properties of FCNF were investigated, and the optimized reaction parameters were explored.

### 2.3. Transmission Electron Microscopy (TEM)

For TEM measurement of FCNF, 0.03% FCNF suspension was ultrasonically dispersed for 20 min and the treated suspension was dropped on a carbon film-coated copper grid and stained with 1.5% phosphotungstic acid. After drying, the morphology and size of FCNFs were observed by a Hitachi-H7650 transmission electron microscope (Hitachi, Ltd., Tokyo, Japan) at an accelerated voltage of 100 kV.

### 2.4. Surface Chemical Structure

After freeze drying, FCNF powder was characterized by Fourier transform infrared (FTIR) and ^13^C nuclear magnetic resonance (^13^C NMR). FTIR spectra were recorded on a Nicolet 380 FTIR spectrometer (Thermo electron Instruments Co., Ltd., Madison, WI, USA) in wave numbers ranging from 4000 cm^−1^ to 400 cm^−1^, with a resolution of 4 cm^−1^. ^13^C NMR spectra were collected at a magic angle spinning rate of 5 kHz, with a proton resonance frequency of 125 MHz.

### 2.5. X-ray Diffraction (XRD)

The crystalline structure of FCNF was characterized by X-ray diffraction (Shimatzu diffractometer, XRD 6100, Kyushu, Japan) with Cu Kα radiation at the wavelength 1.5406 Å. The continuous scanning angle was 6–90° at a scan rate of 0.1 s^−1^. The crystallinity index (*C_r_I*) of FCNF was calculated according to the following equation:

(1) $$C_{r}I=\frac{I_{200}-I_{am}}{I_{200}}\times 100\%$$

where *I*_200_ is the intensity of the peak at 2*θ* about 22°, representing the crystalline region and amorphous region of cellulose. *I_am_* is the intensity of the amorphous background at 2*θ* about 18° [37].

### 2.6. Thermogravimetric Analyzer (TG)

The thermal stability of FCNF was characterized by a thermogravimetric analyzer (NETZSCH STA 449 F3 Jupiter^®^, Selb, Germany) under heating from 25 °C to 700 °C at 10 °C min^−1^ with a flow N_2_ of 20 mL min^−1^ as the protecting gas.

### 2.7. Fluorescent Properties Test

Fluorescent spectra were measured by an RF-5301PC fluorescence spectrometer with 1.0 cm quartz cells at slits of 5/5 nm. The emission wavelength ranged from 300 nm to 650 nm, with a scanning frequency of 40 Hz at a rate of 240 nm min^−1^. After scanning three times in parallel, the optimal excitation wavelength and emission wavelength of FNCF were obtained.

### 2.8. Ionic Sensitivity Tests

After preparing 10 mM Na^+^, ClO_4_^−^, Cl^−^, Li^+^, NO_3_^−^, SO_3_^2−^, OH^−^, K^+^, and Ca^2+^ solutions, 5 mL of the solutions was respectively dispersed in 4 mL of FCNF suspension for the samples containing different ions. The sensitivity of FCNF to different ions was analyzed by characterizing the fluorescence intensity of samples containing different ions, as described in Section 2.8, and the correlation was shown as the following equation [38,39]:

(2) $$I_{0}/I_{H}=K[X]+K[H^{+}]+1$$

where *I*_0_ is the fluorescence intensity in the absence of specified ions; *I_H_* is the fluorescence intensity in the presence of specified ions. The ratio *I*_0_*/I_H_* represents the fluorescence quenching rate. K represents the sensitivity of FCNF to the specified ions. [*X*] and [*H*^+^] are the concentrations of specified ions and hydrogen ions in the solution, respectively. According to the formula, there is a linear relationship between *I*_0_*/I_H_* and [*X*] at a fixed pH (i.e., the (K[*H*^+^] + 1) term becomes a constant). Moreover, the ionic sensitivity K of FCNF can be obtained from the slope of the plot of *I*_0_*/I_H_* vs. [*X*].

## 3. Results and Discussion

### 3.1. Fluorescence Properties

**Figure 1:**
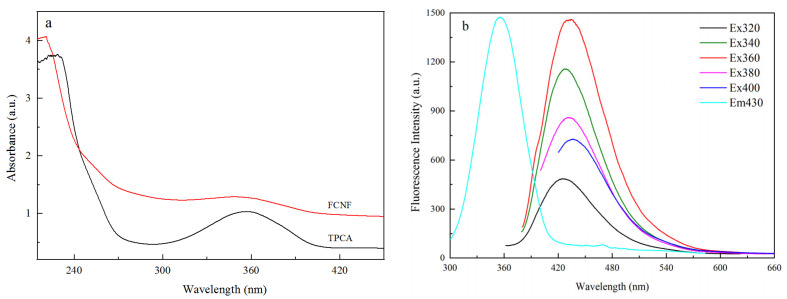
UV–vis spectra of FCNF and TPCA (**a**) and the fluorescence emission spectra of FCNF (**b**).

It can be observed from the UV–vis spectra of FCNF and TPCA in Figure 1a that they have similar absorption peaks, in which the peaks near 225 nm are attributed to the π→π* transition and the wide absorption peaks near 360 nm are attributed to the n→π* transition of the conjugate structure of TPCA [40,41]. This indicated that FCNF was successfully surface grafted with TPCA groups. Figure 1b shows that the fluorescence intensity of FCNF increased significantly as the excitation wavelength (320–360 nm) increased. When the excitation wavelength exceeds 360 nm, the fluorescence intensity shows a decreasing trend. Moreover, the maximum fluorescence intensity is at the excitation wavelength of 360 nm, and the maximum fluorescence emission wavelength is always maintained at around 430 nm, indicating that FCNF has good fluorescence emission performance, independent of the excitation wavelength.

### 3.2. Effect of Reaction Temperature on Yield and Fluorescence Properties of FCNF

**Figure 2:**
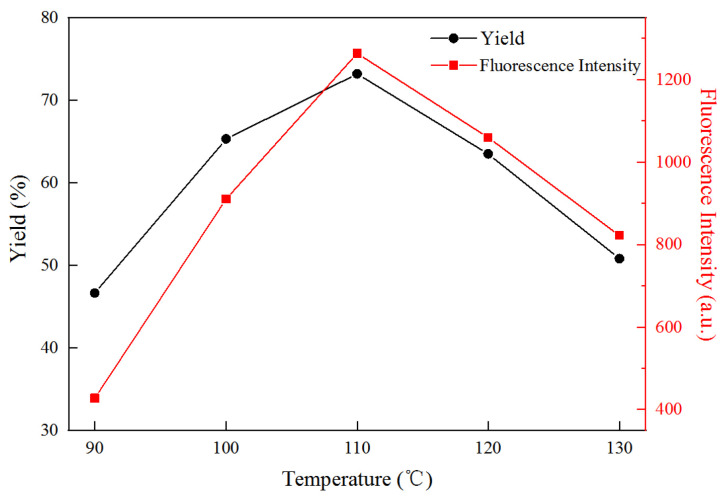
The yield and fluorescence intensity evolution of FCNF with different temperatures.

As shown in Figure 2, the yield and fluorescence intensity of FCNF increased gradually as the reaction temperature increased from 90 °C to 110 °C, under the conditions of a reaction time of 5 h and microwave power of 500 W. The reason for this phenomenon is that the higher reaction temperature is conducive to the diffusion of citric acid into cellulose and the acceleration of mass transfer, which promotes the hydrolysis of the amorphous region of cellulose, and the dehydration reaction of citric acid and L-cysteine, resulting in a significant increase in the yield and fluorescence intensity of FCNF. When the reaction temperature increased to 110 °C, the yield of FCNF reached 73.2%, with the maximum fluorescence intensity. As the temperature continued to rise, the hydrolysis reaction of cellulose was intensified, resulting in the destruction of the crystalline region and excessive hydrolysis of the cellulose into glucose. In addition, the dehydration reaction of citric acid and L-cysteine was also violent with an increasing reaction temperature, leading to the production of by-products. Therefore, an excessively high reaction temperature reduced the yield and intensity of FCNF, and 110 °C was considered to be the optimal reaction temperature, owing to its maximum yield of FCNF in this experiment.

### 3.3. Effect of Reaction Time on Yield and Fluorescence Properties of FCNF

**Figure 3:**
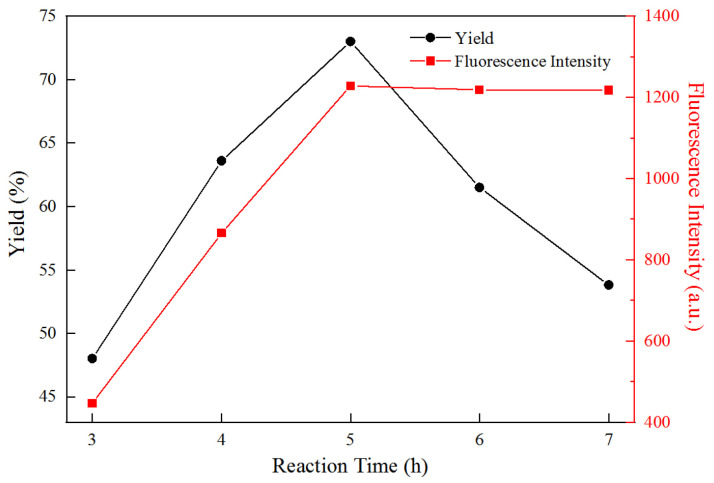
The yield and fluorescence intensity evolution of FCNF with different times.

Figure 3 shows that under a reaction temperature of 110 °C and microwave power of 500 W, the yield and fluorescence intensity of FCNF increased with the increase in reaction time from 3 h to 5 h. This may be because at a certain reaction temperature, prolonging the reaction time is conducive to the full hydrolysis of the cellulose amorphous region, without destroying the crystallization region. In addition, it is conducive to the dehydration reaction of citric acid and L-cysteine, which improves the graft rate of TPCA on the surface of nanocellulose, resulting in an increase in the FCNF yield and fluorescence intensity. In addition, the increase in reaction time can promote the dehydration reaction of citric acid and L-cysteine, and improve the graft rate of TPCA on the nanocellulose surface. The maximum yield of 73% and maximum fluorescence intensity of FNCF were achieved at 5 h. When the reaction time exceeded 5 h, the yield decreased gradually and the fluorescence intensity remained stable, with the color of FCNF deepening from light yellow to dark yellow. The reduction in the yield is mainly due to the excessive hydrolysis of cellulose, while the constant fluorescence intensity is attributed to the dynamic equilibrium of the dehydration reaction of citric acid and L-cysteine within a certain time, leading to there being no further increase in the graft rate with the prolonged reaction time.

### 3.4. Morphology

**Figure 4:**
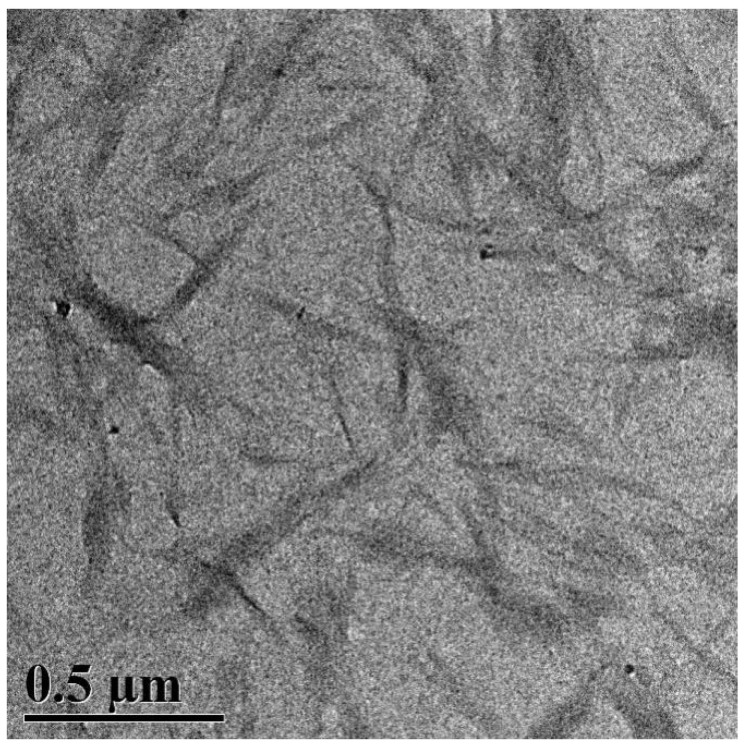
TEM images of FCNF.

The morphology of FCNF was observed by TEM imaging, as is shown in Figure 4. As is shown in the images, short rod-like shapes, with a length of 200–300 nm, a diameter of 10–20 nm, and an aspect ratio of 20–30, were observed. It was indicated that, under microwave-hydrothermal conditions, cellulose was hydrolyzed by citric acid, the supramolecular structure of cellulose was depolymerized, the amorphous region was destroyed, and cellulose nanofibers (CNFs) were formed. Furthermore, FCNF was fabricated by the dehydration reaction between CNF and TPCA. The short rod-like FCNF interweaves into a network structure, which enables it to play a reinforcing role in the construction of nanocomposites.

### 3.5. FTIR

**Figure 5:**
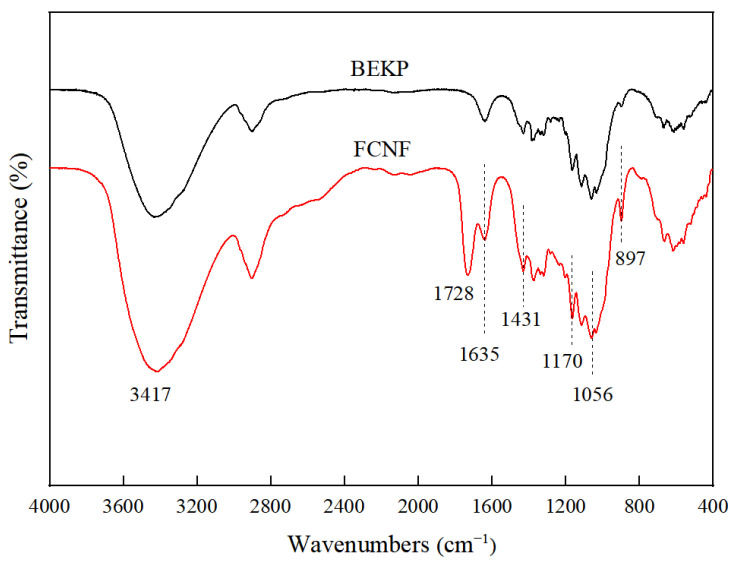
FTIR spectra of FCNF.

Obvious characteristic peaks of the cellulose I crystalline structure, belonging to FCNF and BEKP, can be observed in Figure 5, at 1635, 1431, 1170, 1056, and 897 cm^−1^, indicating that the conformation and skeletal structure of FCNF after hydrolysis and Fischer esterification do not change, compared with BEKP [42]. The peak at 1431 cm^−1^ is attributed to the symmetric bending vibration of CH_2_, and represents the absorption band of the crystalline region [43]. The absorption peaks at 1056 cm^−1^ and 1114 cm^−1^ can be assigned to the C-O stretching vibration and skeletal vibration of the glucopyranose ring, respectively. Compared with BEKP, the peak intensities at 1056 cm^−1^ and 1114 cm^−1^ increased, indicating an increase in the content of FCNF crystalline regions. The higher intensity of the peak at 3417 cm^−1^ in the FCNF spectrum than that of BEKP is due to the hydrolysis of the amorphous region, which exposes more hydroxyl groups. Differing from BEKP, the new absorption peak at 1728 cm^−1^ of FNCF belongs to the stretching vibration of C=O in the ester group [44], indicating that FCNF is formed by the simultaneous hydrolysis and esterification of cellulose. Therefore, FCNF was successfully surface grafted with TPCA groups and maintained the basic structural unit of cellulose.

### 3.6. ^13^C NMR Analysis

**Figure 6:**
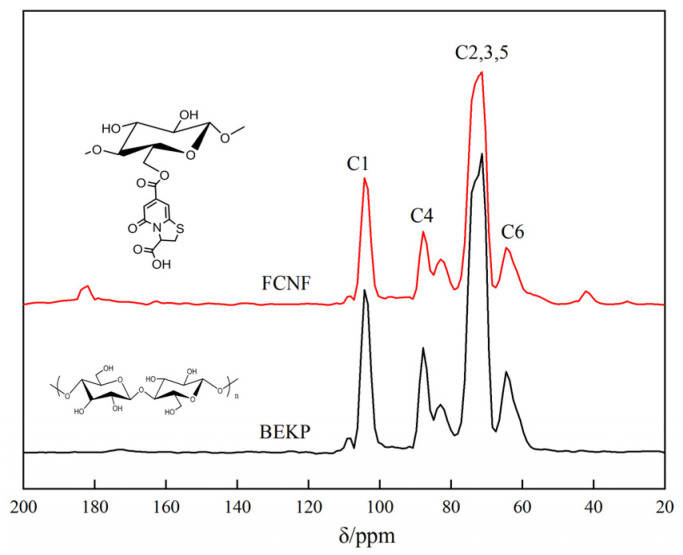
^13^C NMR spectra of FCNF.

^13^C NMR was used to determine the surface chemical structure of FNCF (Figure 6). The spectra of FCNF and BEKP both displayed typical signals from the cellulose I crystalline structure, which are assigned as follows: C1 (104.1 ppm), C2/C3/C5 (71.7 ppm, 74.1 ppm), C4 (87.7 ppm), and C6 (64.2 ppm) peaks belong to carbons of the glucopyranose rings in the crystalline regions [45], whereas C4 (82.7 ppm) and C6 (62.5 ppm) peaks are attributed to the carbons of the glucopyranose rings in the disordered regions [46]. In the spectrum of FNCF, the appearance of peaks at about 41.8 and 182.5 ppm are assigned to the resonance absorption of carbons in citric acid [47,48], and the peaks at about 30.5 and 162.8 ppm are the contribution of carbons in the TPCA group, which indicated that the TPCA group was successfully immobilized on FCNF by esterification with the hydroxyl group of cellulose nanofibers.

### 3.7. XRD

**Figure 7:**
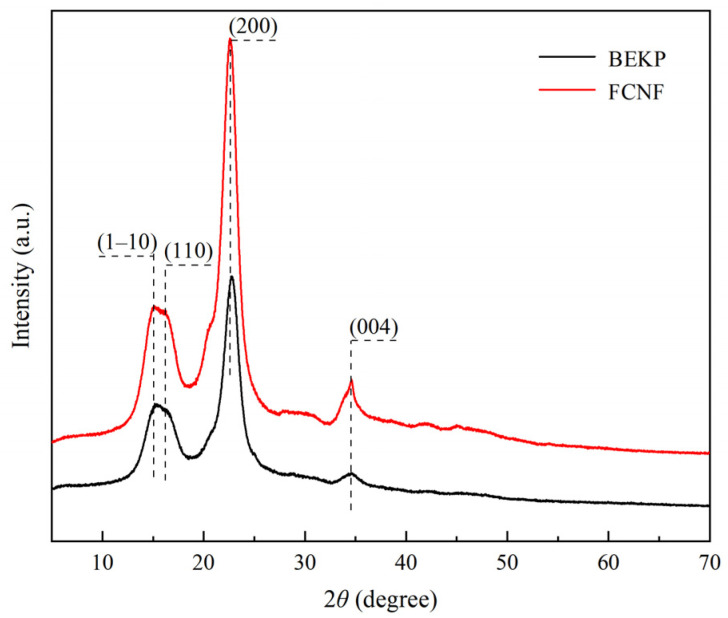
XRD patterns of FCNF.

In the XRD spectra of FCNF and BEKP (Figure 7), the diffraction peaks at 2*θ* = 15.1°, 16.3°, 22.5°, and 34.4° correspond to (1–10), (110), (200), and (004) planes of cellulose I_β_ crystals, respectively, indicating the preservation of the cellulose crystal structure during one-pot preparation of FCNF [49]. The inherent crystal structure of FCNF remained stable in the reaction, as an insignificant change occurs between the diffraction peaks of BEKP and FNCF, but the enhancement of peak intensity at the (200) plane of FNCF implies a more complete crystal structure of FNCF. The crystallinity of FCNF (80%) is significantly higher than that of BEKP (64%), mainly due to the hydrolysis of the amorphous regions to form FCNF with higher molecular regularity. In addition, the high crystallinity and nano size effect of FNCF can effectively improve the mechanical performance of composites, and have broad application potential in enhancing the properties of biomass nanocomposites [50].

### 3.8. TG

**Figure 8:**
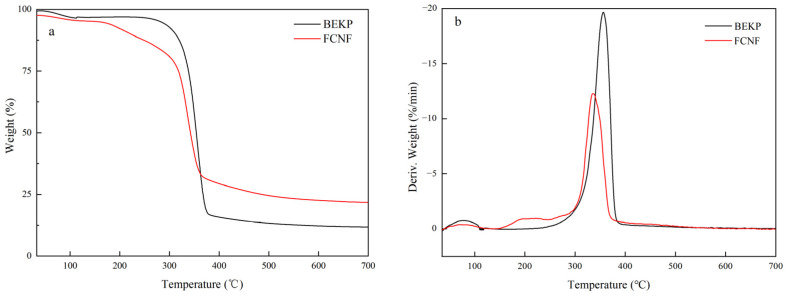
(**a**) TG and (**b**) DTG curves of FCNF.

It can be observed from the TG/DTG curves of FCNF in Figure 8 that the thermal decomposition of FCNF consists of the following three stages: the evaporation of free water on the surface of FCNF (<115 °C), the thermal decomposition of the glucopyranose ring, resulting in a sharp decline (290–400 °C), and the carbonization of the remaining products (>400 °C). As shown in the DTG curves of BEKP (Figure 8b), the onset temperature of thermal decomposition and the temperature at the maximum weight loss rate are 307 °C and 356 °C, respectively. Compared with BEKP, the thermal stability decreased with an initial decomposition temperature of 297 °C and the temperature at the maximum weight loss rate of 336 °C. The main reason for this is related to the introduction of the TPCA group, whose -COOH can lead to the generation of glucuronic acid, and it will then be sensitive to heat, so as to be thermally degraded at lower temperatures [51]. Additionally, the smaller particle size of FCNF exposes a larger surface area and accelerates the thermal decomposition of FCNF, resulting in a decrease in its thermal stability [52]. Moreover, the thermal stability of FCNF is better than that of nanocellulose prepared by the traditional sulfuric acid method and TEMPO oxidation method, which can be attributed to the higher crystallinity and slower heat transfer rate of FCNF, thus improving its heat resistance to a certain extent [53].

### 3.9. Responsiveness of Chloride Ions

**Figure 9:**
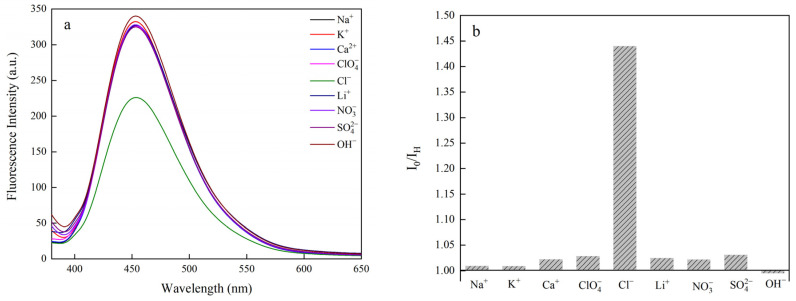

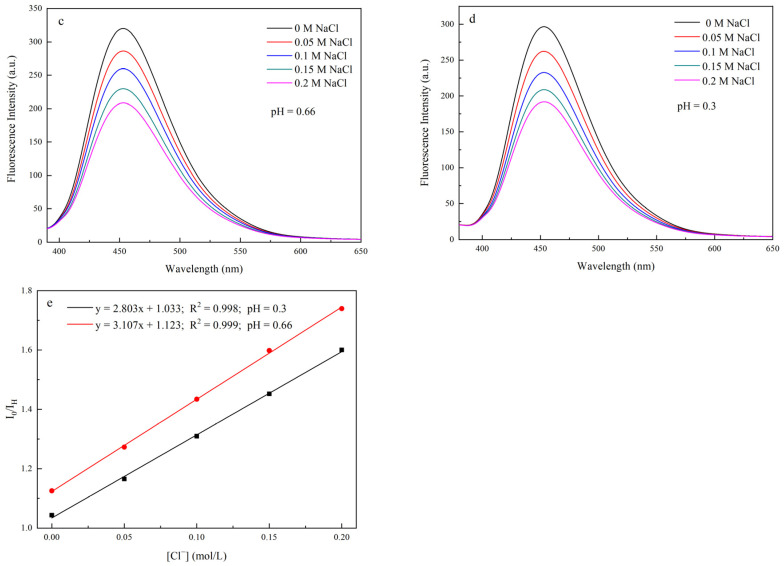
Ion response detection of FCNF. (**a**) The emission spectrum of FCNF solution with different ions; (**b**) the change in fluorescence intensity before and after (Ex = 360 nm); the effect of chloride ion concentration on the fluorescence intensity of FCNF when (**c**) pH = 0.66 and (**d**) pH = 0.3; (**e**) fitting regression curve of FCNF to the fluorescence quenching of chloride ion.

Fluorescence emission spectra of FCNF (Ex = 360 nm) were obtained by adding solutions containing different ions in the same volume and concentration (1 mg/mL) into the suspension of FCNF, with pH 2, as is shown in Figure 9a. The fluorescence intensity of the solution containing Cl^−^ is the lowest among them. Figure 9b shows that the fluorescence quenching rate of Cl^−^ on FNCF reached a maximum of 74.2% under a certain pH condition. The sensitivity of FCNF to Cl^−^ stems from the reduction in electrostatic repulsion of carboxyl and carbonyl groups during each continuous protonation process in their respective pH regions, enabling the chlorine complex to form in the excited state, changing the nature and rate of the nonradiative transition process that competes with the luminescence process, stimulating the gradual quenching of chloride under acidic conditions [54,55]. To further explore the effect of pH on the Cl^−^ responsive properties of FCNFs, we designed a simple experimental model. Figure 9c,d show the fluorescence emission spectra (Ex = 360 nm) of FCNF, adding 1 mL Cl^−^ solution with different concentrations at pH = 0.66 and pH = 0.3, respectively. It can be observed from the figures that the fluorescence quenching rate of Cl^−^ on FCNF is accelerated with the decline in the pH value. Fitting the regression curve of FCNF to the fluorescence quenching of the chloride ion was undertaken to more clearly and intuitively reflect the influence of Cl^−^ concentration and pH on the fluorescence quenching of FCNF (Figure 9e). The result shows that the fluorescence quenching of FCNF by Cl^−^ responds linearly at the concentration of 0.2 M, and the fluorescence quenching efficiency is higher at a lower pH, indicating that the fluorescence quenching behavior of FCNF is attributed to the interaction of excited-state ions and chloride under acidic conditions, which leads to partial charge transfer and spin–orbit coupling. Therefore, FCNF has high sensitivity to Cl^−^ under acidic conditions, and can be used for the quantitative analysis and detection of Cl^−^ content, which has potential application value in chemical sensing, biosensing, and other fields.

## 4. Conclusions

Based on the principle of the microwave-hydrothermal reaction, FCNFs with a high yield were prepared by the one-pot method in the aqueous phase, which avoided the tedious separation process of intermediate products and the use of organic solvents. This strategy improved the reaction efficiency and yield to realize the green, low-carbon and efficient preparation of FCNF. The yield of FCNF reached 73.2% under the conditions of a microwave power of 500 W, reaction temperature of 110 °C, and reaction time of 5 h. It was found that FCNF has good dispersion stability in water, with a length of 200–300 nm, a diameter of 10–20 nm, an aspect ratio of 20–30, and crystallinity of 80%. Furthermore, FCNF has stable fluorescence performance and good sensitivity to Cl^−^, which can be used for the quantitative detection of chloride ions, indicating that it has broad application prospects in the fields of fluorescent labeling, biomedicine, sensing, and detection.

## Data Availability

The data presented in this study are available on request from the corresponding author.
